# The Development and Progress of Health Information Technology in Iran

**DOI:** 10.1002/hsr2.71106

**Published:** 2025-07-23

**Authors:** Azita Balaghafari, Hasan Siamian

**Affiliations:** ^1^ Department of Health Information Technology, School of Allied Medical Sciences Mazandaran University of Medical Sciences Sari Iran; ^2^ Department of Health Information Technology, School of Allied Medical Sciences, Health Sciences Research Center Mazandaran University of Medical Sciences Sari Iran

**Keywords:** curriculum, delivery of health care, health information technology, Iran, knowledge management, medical informatics, medical records, students, universities

## Abstract

**Background and Aims:**

Health information technology (HIT) is a vital field within medical science that emerged in the early 2000s to meet the growing demand for IT solutions in healthcare. This article aims to provide an overview of HIT, focusing on the educational programs available, their enrollment capacity across various courses, and the advancements in HIT within Iranian medical universities from its inception to the present day.

**Methods:**

The study compiles comprehensive information regarding the educational programs, detailing their components, historical development, including past curriculum units, establishment years of different university degrees, and student enrollment capacities across Iranian universities.

**Results:**

The findings outline the characteristics of the curriculum, including unit details and semester structures within the School of Allied Medical Sciences. Additionally, it highlights the development and progress of educational programs, past curricula, course units, establishment years of various university courses, and student enrollment capacities nationwide.

**Conclusion:**

This study sheds light on the development of HIT in Iran, which began in the 1980s in response to the increasing need for IT in healthcare. The transition from paper‐based medical records to electronic systems in the 1990s marked a significant shift, leading to the rebranding of medical documentation as HIT. This transition has established a modern framework for knowledge management and secure information exchange in the health sector. The article defines HIT, discusses its background, and provides an overview of educational processes along with changes and diversity in course offerings and student attraction within Iranian medical universities. This perspective has been developed over many years.

## 1 Introduction

Health information technology (HIT) is a branch of medical sciences that uses computer hardware and software to process information, store, retrieve, and share them, and provides the possibility of using health data and knowledge to facilitate communication and decision‐making [1]. Rapid advancements in information technology have laid the basis for the move to information management job positions and health informatics education. In fact, information technology becomes an essential component of different healthcare educational programs worldwide. However, scholarly research about health informatics, particularly in developing countries, is still limited [2]. HIT is an interdisciplinary course based on medical sciences, information technology, and management. The graduates of this field will be able to take charge and manage the departments related to information technology and the units of handling and supervision of insurance in all institutions providing health and medical services, as well as in the field of carrying out organizational processes using information technology, which in this field for health and health care are used, play a role [3].

Information technology also supports the management of health information across computer systems and the secure exchange of health information between users, providers, payers, and quality monitors [4]. Today, clinics and hospitals are completely dependent on information technology to redesign the entire health care process [5]. The end users of this technology include patients, doctors, other health care providers, medical researchers, health care insurance companies, quality assurance and regulatory bodies, pharmaceutical and medical equipment companies, and so forth. Since these entities assume a wide range of roles and have diverse needs and goals, information technology and the systems that underlie it are critical to the delivery and advancement of health care at a societal scale [6, 7, 8]. The health care system helps to treat diseases, increase life and improve the welfare of communities by using electronic health records (EHR), personal health records, electronic prescribing (E‐prescribing), etc [9]. Despite the many benefits that HIT systems provide, if not properly planned, designed, implemented, and managed, new technology poses new and unforeseen risks to healthcare quality and patient safety.

HIT has the potential to improve healthcare delivery and engagement [10]. More effective use of HIT, including EHRs… and care [11]. Studies from as early as the 1990s have shown that EHRs and associated clinical decision support tools have potential in helping with patient care and population health needs [12]. On the other hand, obstacles like clinician readiness [13] and clinical workflow integration [14] impede EHRs' full benefits. Enabling widespread interoperability—the ability of HIT systems to exchange information and to use that information without special effort—is a primary focus of public policy on HIT. More information on clinicians' experience using that technology can serve as one measure of the impact of that policy [15]. Despite many benefits offered by HIT systems, new technology brings new and unforeseen risks to healthcare quality and patient safety if they're not properly planned, designed, implemented, and managed [16]. Although intended to support improvement, the rapid adoption and evolution of technologies in health care can also bring about unintended consequences related to safety [17].

HITs have some benefits and may have some potentially negative impacts. Therefore, it is difficult to plan for future HITs [18, 19, 20]. HIT includes a variety of information and communication technologies which are used to collect, transmit, display, or store patient data [21, 22].

After establishing the critical role of HIT in enhancing healthcare delivery and outcomes, it is imperative to emphasize that the HIT curriculum must undergo continuous evolution. This evolution is essential not only to address the immediate challenges faced by Iran's healthcare system but also to anticipate and prepare for future developments. The dynamic nature of healthcare—shaped by rapid technological advancements, changing patient demographics, and shifting policy landscapes—demands that educational programs remain responsive and relevant. By aligning the HIT curriculum with both contemporary needs and emerging trends, we can ensure that future professionals are equipped with the knowledge and skills necessary to navigate an increasingly complex healthcare environment. This alignment will ultimately contribute to improved patient care, greater efficiency in health services, and enhanced public health outcomes across Iran. Discuss previous studies that have examined the relationship between HIT education and healthcare outcomes, emphasizing the need for curricula to adapt to changing healthcare landscapes.

Therefore, the use of information technology in the provision of health care requires a trained and capable workforce [16]. The purpose of this article is to introduce the field of health information technology (HIT) in Iran, providing an overview of the development and progress of HIT educational programs and student enrollment capacities in various courses. Additionally, the article investigates the evolution of HIT within Iranian medical universities from its inception until the present time.

## 2 Methods

The research titled “The development and progress of HIT in Iran” is a historical analysis and literature review approach, conducted through a review of library and internet resources regarding the delivery of courses. This study focuses on the training of undergraduate students at the Sari Paramedical School and the curriculum emphasis in higher education institutions across the country. It specifically examines the approved educational programs for bachelor's degree in HIT, as ratified in the 40th session of the Supreme Council for Medical Sciences Planning on August 20, 2009, and in the 73rd session on July 13, 2019 [23].

Identification of Sources: A wide range of sources was identified, including National Entrance Exam Booklets of the Iranian Educational Assessment Organization, official reports from the Supreme Council of Medical Sciences, government publication, Persian books, and educational pamphlets related to HIT in Iran.

To analyze the development and progress, the study addresses general characteristics of the educational program, details of program units, historical development of course education (including curricula and course units from previous years), as well as information on the establishment years of various academic levels and student admission capacities across universities in Iran.

## 3 Results

- Specifications of the training program of the field

The general characteristics of the continuing undergraduate education program in the field of HIT are given in Table 1.

**Table 1:** Key features of the continuing undergraduate education program in health information technology.

Sequence	Title/Section	Description
1	Introduction	Health is the most basic human need and a fundamental right. Health information technology (HIT) is essential for advancing public health, enabling effective planning and decision‐making, and supporting healthcare transformation through innovations such as electronic health records and integrated health systems. This curriculum revision is based on comprehensive needs assessments involving stakeholders nationwide. The program aims to train skilled HIT professionals to meet the evolving needs of the healthcare sector.
2	Course title	Health information technology
3	Degree level	Bachelor of Science (B.Sc.)
4	Admission requirements	Admission is through the National Organization for Educational Testing in Iran.
5	Job opportunities for graduates	Graduates can work in: – Hospitals – Treatment deputy offices – Health deputy offices – Research deputy offices – Other administrative areas – Specialist roles in relevant educational groups
6	Mission	To train knowledgeable, capable, responsible, and health‐conscious professionals in HIT, specializing in the application of IT in healthcare, processing and managing information, facilitating communication among healthcare teams, and supporting decision‐making. Graduates will provide essential clinical and financial information and uphold the rights of patients and hospitals.

Table 1 indicates that:

The educational program outlined in the table, approved by the 73rd session of the Supreme Council of Medical Sciences Planning on April 23, 2018, reflects a comprehensive approach to addressing the critical need for trained professionals in HIT.

1. Importance of HIT: The introduction emphasizes that health is a fundamental human need and a universal right, highlighting the role of HIT as a vital tool for enhancing public health. This underscores the necessity of integrating technology into healthcare to facilitate effective planning and decision‐making.
2. Curriculum Development: The revision of the curriculum for the bachelor's degree in HIT is based on thorough needs assessments involving various stakeholders, including students, employers, and experts. This participatory approach ensures that the curriculum remains relevant and aligned with current industry demands.
3. Focus on Practical Skills: The program aims to equip graduates with both professional knowledge and practical skills necessary for effective decision‐making and problem‐solving in health information management. This focus on hands‐on training is crucial for preparing students to meet real‐world challenges in healthcare settings.
4. Job Readiness: The table outlines potential job positions for graduates, indicating a clear pathway for employment in various sectors such as hospitals, treatment and health deputy offices, and research departments. This clarity in career prospects enhances the program's appeal to prospective students.
5. Mission Statement: The mission of the program is well‐defined, aiming to produce knowledgeable and responsible professionals who can effectively utilize information technology in healthcare settings. This mission aligns with global trends toward digital health solutions and emphasizes the importance of communication within healthcare teams.
6. Alignment With National Health Policies: By addressing the requirements set forth in the health system transformation document, this program not only responds to immediate educational needs but also contributes to broader national health objectives. This alignment is essential for fostering a workforce capable of driving improvements in public health.
7. Future Prospects: The program's emphasis on developing capable and skilled experts in HIT positions graduates to play a significant role in advancing healthcare delivery systems. As technology continues to evolve, the ability of graduates to adapt and innovate will be critical for enhancing patient care and operational efficiency within healthcare organizations.

In summary, this educational program is designed to meet the growing demands of the healthcare sector by providing a robust curriculum that combines theoretical knowledge with practical skills, ensuring that graduates are well‐prepared to contribute effectively to the field of HIT.

The details of the course units for the HIT program are presented in Table 2.

**Table 2:** Detailed overview of educational program units in health information technology.

The educational program approved by the 73rd session of the Supreme Council of Medical Sciences Planning dated April 23, 2018			
Duration of the course and its structure: 4 years (according to the educational regulations of continuous undergraduate courses approved by the Supreme Planning Council of Medical Sciences)			
The total number of course units		General courses	24 units
		Basic courses	21 credits
		Units of specialized courses	69 units
		Internship and training in the field of	16 units
		Units in total	130 units
Course units	Theoretical foundations of Islam (4 units), Islamic ethics (2 units), Islamic revolution (2 units), Islamic history and civilization (2 units), familiarity with Islamic sources (2 units), Persian literature (3 units), general English language (3 units), physical education 1 (1 unit), physical education 2 (2 units), family and population knowledge (2 units), history and culture and civilization of Islam and Iran (2 units), physiology (3 units), anatomy (2 units), introduction to pathogenic agents (2 units), pharmacology (2 units), basic mathematics (2 units), basic vital statistics (2 units), basics of epidemiology (2 units), computer basics (1 unit), computer basics laboratory (1 unit), research method (2 units), principles of management (2 units), medical terminology 1 (2 units), medical terminology 2 (2 units), pathology 1 (2 units), pathology 2 (2 units), pathology 3 (2 units), surgical procedures, diagnostic services, treatment and medical consumables (2 units), inferential vital statistics (2 units), HIT specific language (3 units), HIM specific language (3 units), indicators and health data analysis (2 units), basics of accounting and health economics (2 units), performance improvement management in health and treatment centers (2 units), processing and calculation of hospital insurance documents (2 units), processing and calculation of outpatient insurance documents and Paraclinic (2 units), how to monitor and inspect medical centers (1 unit), health information management 1 (2 units), health information management 2 (2 units), disease coding 1 (2 units), disease coding 2 (2 units), disease coding 3 (2 units), medical procedure coding (1 unit), specialized international classifications (2 units), mortality coding (2 units), familiarity with applications (1 unit), applications laboratory (1 unit), introductory programming (1.5 units), introductory programming laboratory (0.5 units), data structures (2 units), advanced programming (1.5 units), advanced programming laboratory (0.5 units), computer networks (1.5 units), computer networks laboratory (0.5 units), databases (1.5 units), database laboratory (0.5 units), health information systems (2 units), management of health information systems (2 units), application of health information systems (2 units), application laboratory of health information systems (1 unit), analysis and display of health data (1.5 units), analysis and display of health data laboratory (0.5 units), research project (2 units), 1 and 2 health information management internship (2 units), health data analysis and indicators internship (1 unit), coding internship (1 unit), health information systems application internship (1 unit), mortality coding internship (1 unit), internship in field 1 (5 credits), and internship in field 2 (5 credits). Total: 130 units		

Table 2 outlines the educational program for the bachelor's degree in HIT, which was approved by the 73rd session of the Supreme Council of Medical Sciences Planning on April 23, 2018. The program is structured to be completed over a duration of 4 years, adhering to the educational regulations for continuous undergraduate courses established by the Supreme Planning Council of Medical Sciences.

Course Structure

The total number of course units required for graduation is 130, which includes:

– General courses: 24 units;
– Basic courses: 21 units;
– Specialized courses: 69 units;
– Internship and training: 16 units.

The table details the specific courses included within these categories, emphasizing a comprehensive curriculum designed to equip students with both theoretical knowledge and practical skills essential for a career in HIT.

Course Units Breakdown

The course units are categorized as follows:

1. General Courses: These include foundational subjects such as Islamic studies, Persian literature, physical education, and basic sciences like physiology and anatomy. These courses aim to provide students with a well‐rounded educational background.
2. Basic Courses: This section covers essential topics such as pharmacology, epidemiology, computer basics, and medical terminology. These courses are crucial for building the foundational knowledge necessary for specialized training.
3. Specialized Courses: A significant portion of the curriculum focuses on specialized HIT topics, including health information management, disease coding, health data analysis, and health information systems. This section prepares students for the specific challenges they will face in their careers.
4. Internships: The program includes practical training through various internships that allow students to apply their knowledge in real‐world settings. This hands‐on experience is vital for developing competencies required in the field.

Overall, Table 2 presents a structured and detailed overview of the educational program for HIT, highlighting its comprehensive nature and alignment with industry needs. By integrating general education with specialized training and practical experience, the program aims to produce qualified professionals ready to contribute effectively to the healthcare sector.

- History of the development of HIT education in Iran

The General Department of Medical Services of the Ministry of Health in 1965 in the Organization of Newly Established Hospitals determined a medical file for each hospital and recognized the need to establish a department of medical records and record keeping for patients, and published a guide for preparing medical records in the same year. At the same time as these developments, the existence of trained and educated employees in this field was recognized as necessary, and to fill this gap and restore the technical organization of hospitals, the Ministry of Health established a school for the first time in 1968, which taught in the fields of assisting radiology technicians, pharmacy and medical records. The course duration was only one year, and applicants were required to have at least a high school diploma. In addition to studying theoretical courses, after internship in educational hospitals, students were practically familiar with the characteristics of this technique and its variety of activities. Red Crescent Technical Training Center (formerly The Red Lion and Sun Society) organized 6‐month courses of medical documents since 1973, and after the end of the course, each trained person was sent to one of the hospitals of the cities affiliated to Red Crescent as the person in charge of medical documents. Some of the courses of this course are: Medical records, disease coding, hospital statistics, archival principles, public health, anatomy, medical terminology, and internship.

The evolutionary course of undergraduate curriculum of the field of HIT in Iran by section, the name of the field based on the educational documents and titles available from the 1350s = 1971s until now, with the titles and number of course units for introduction and familiarization with the structure of the composition of course units as described in Table 3 is presented.

**Table 3:** Curriculum overview of associate and bachelor courses in Iran: A historical perspective.

Associate degree	Medical records keeper	1351–1971	Principles of health (2 units), principles of archiving (2 units), principles of microfilm coding and archiving (3 units), principles of statistics and communication (3 units), economics (2 units), anatomy and physiology (2 units), English (4 courses, each 3 units), total (12 units), history of culture (2 units), sociology (3 units), biology (2 units), psychology (3 units), Persian and writing (two 4‐unit courses) (8 units), internship (two lessons of 3 units) (6 units), typing (two lessons of 2 units and two lessons of 3 units) (10 units), social worker (3 units), management of health services (2 units), medical terminology (one lesson of 2 units) and one lesson of 3 units (5 units), and exercise (1 unit). Total: 71 units
		1354–1975	Principles of health (3 units), principles of archiving (2 units), principles of statistics (2 units), economics (2 units), anatomy (2 units), physiology (2 units), English (three lessons of 3 units and one lesson of 4 units) (13 units), history of culture (2 units), sociology (3 units), biology (2 units), psychology (3 units), Persian and writing rituals (2 units), Persian (three lessons of 2 units) (6 units), internship (two lessons of 3 units) (6 units), medical degrees (one lesson of 2 units and one lesson of 3 units) (5 units), medical terminology (one lesson of 2 units and two lessons of 3 units) (8 units), microbiology (2 units), family planning (1 unit), medical law (2 units), and demography (1 unit). Total: 69 units
		1357–1978	Principles of public health (3 units), principles of public archives (2 units), principles of statistics (2 units), vital statistics (2 units), economics (2 units), anatomy (2 units), physiology (2 units), English (1 course of 2 units and two courses of 3 units and one course of 4 units (12 units), history of culture (2 units), sociology (3 units), biology (2 units), psychology (3 units), Persian and writing system (2 units), internship (one lesson of 3 units and one lesson of 4 units) (7 units), principles of management and human relations (2 units), medical terminology (one lesson of 2 units and two lessons of 3 units) (8 units), sports (1 unit), medical law (2 units), medical documents (one 2‐unit lesson and one 3‐unit lesson) (5 units), hospital organization and management (2 units), family planning (1 unit), demography (1 unit), and microbiology (2 units). Total: 70 units
	Recording of medical statistics and documents	1361 = 1982	Persian (text, grammar, and writing system) (4 units), Arabic (pronunciation, syntax, and reading) (2 units), foreign language (2 units), Islamic history (2 units), basic mathematics and basics of statistics (3 units), physical education (1 unit), environmental health (2 units), medical sociology (2 units), medical law (2 units), medical ethics (2 units), hospital management (2 units), vital statistics (3 units), computer and its application (3 units), general medicine (3 units), social work (2 units), first aid (3 units), typing (2 units), statistical evaluation and analysis (3 units), computer (Fortran language) (3 units), special language (4 units), medical terms (3 units), medical documents 1 (2 units), medical documents 2 (2 units), principles of archiving 1 (2 units), principles of archiving 2 (2 units), coding of diseases (3 units), seminar on hospital issues and medical records (2 units), internship 1 (6 units), and internship 2 (6 units). Total: 82 units
		1366–1987	Persian (2 units), foreign language (2 units), Islamic education 1 (2 units), Islamic education 2 (2 units), ethics and Islamic education (2 units), basic mathematics and basics of statistics (3 units), physical education (1 unit), environmental health (2 units), medical sociology (2 units), medical law (2 units), medical ethics (2 units), hospital management (2 units), vital statistics (3 units), computer and its application (3 units), general medicine (3 units), social work (2 units), first aid (3 units), typing (2 units), statistical evaluation and analysis (3 units), computer (Fortran language) (3 units), specialized language (4 units), medical terms (3 units), medical documents 1 (2 units), medical documents 1 (2 units), medical documents 2 (2 units), principles of archiving 1 (2 units), principles of archiving 2 (2 units), coding of diseases (3 units), seminar on hospital issues and medical records (2 units), internship 1 (6 units), and internship 2 (6 units). Total: 80 units
	Medical records	1369 = 2000	Islamic education 1 (2 units), Persian 1 (2 units), foreign language 1 (practical and theoretical) (2 units), Islamic education 2 (2 units), Islamic ethics and education (2 units), physical education (practical) 1 unit), hospital environmental health (3 units), physiology and anatomy (4 units), familiarity with parasites, fungi and microbiology (3 units), ethics and professional regulations (2 units), introductory psychology and human relations (2 units), basic mathematics (2 units), general medicine 1 (2 units), general medicine 2 (2 units), medical terms 1 (3 units), specialized language 1 (2 units), specialized language 2 (2 units), principles of management Hospital (2 units), computer and its application (4 units), typing (2 units), medical documents 1 (2 units), medical documents 2 (2 units), archiving principles and methods (2 units), medical archiving (2 unit), disease coding 1 (2 units), disease coding 2 (2 units), health indicators and hospital statistics (3 units), preliminary vital statistics 1 (2 units), internship 1 (4 units), internship 2 (4 units), and internship 3 (4 units). Total: 75 units
		1383 = 2003	Islamic theoretical foundations (2 units), Islamic ethics (2 units), Persian (3 units), foreign language (3 units), population and family planning (2 units), physical education (1 unit), epidemiology basics (2 units), physiology (2 units), anatomy (2 units), psychology of human relations (2 units), medical terms (3 units), pathology 1 (3 units), pathology 2 (2 units), specific language 1 (2 units), special language 2 (2 units), principles of management (2 units), familiarity with computers (2 units), basics of information technology (2 units), common systems of classification of diseases and medical procedures 1 (3 units), common systems of classification of diseases and medical procedures 1 (3 units), medical records 1 (2 units), medical records 2 (2 units), organization and storage of health data (2 units), mathematics and statistics (2 Units), indicators and analysis of health data (3 units), internship 1 (4 units), internship 2 (4 units), and internship 3 (4 units). Total: 68 units
Discontinuous bachelor	Medical records keeper	1355 = 1976 1357 = 1978	Vital statistics (2 units), sports (1 unit), general medicine (2 units), mathematics (2 units), English (one lesson of 3 units and two lessons of 2 units), medical documents (2 units), medical terminology (two 3‐unit course), hospital organization and management (2 units), writing code (2 units), principles of computer use in health services (2 units), computer programming principles (2 units), seminar (1 unit), principles of management and relations Humanities (2 units), economics (2 units), health service management (2 units), environmental health (2 units), medical librarianship (2 units), and principles of health insurance (2 units). Total: 43 units
	Recording of medical statistics and documents	1357 = 1978	Economics (2 units), English (3 units), mathematics (2 units), medical records (2 units), medical terminology (3 units), vital statistics (2 units), English (one lesson of 2 units and one lesson of 3 units), seminar (2 units), Farsi and script (2 units), first aid (4 units), disease coding (2 units), medical terminology (3 units), Islamic education (3 units), environmental health (2 units), history of Islamic culture (2 units), public relations (2 units), pharmacology (2 units), general medicine (2 units), medical librarianship (2 units), vaccination (2 units), accounting principles (2 units), environmental health (2 units), health education (2 units), blood transfusion (1 unit), and internship (18 units). Total: 73 units
	Medical records	1369 = 2000	Islamic ethics and education 2 (1 unit), Persian 2 (2 units), physical education 2 (practical) (1 unit), Islamic history (2 units), Islamic revolution and its roots (2 units), Islamic texts (2 units) biology (2 units), pharmacology (2 units), medical sociology (2 units), general medicine 3 (3 units), general medicine 4 (3 units), preliminary vital statistics 2 (2 units), research methodology and ethnology (3 units), computer (Fortran language) (3 units), medical terminology 2 (2 units), medical terminology 3 (2 units), proprietary language 3 (2 units), proprietary language 4 (2 units), legal aspects of documents medicine (2 units), specialized archiving of medical documents (2 units), disease coding 3 (2 units), disease coding 4 (2 units), medical documents 3 (2 units), medical documents 4 (2 units), document statistics seminar medicine and hospital issues (2 units), medical records management (2 units), internship 4 (4 units), internship 5 (4 units), and internship 6 (4 units). Total: 79 units
		1374 = 1995	Islamic education 2 (2 units), Islamic revolution and its roots (2 units), Islamic history (2 units), Islamic texts (teaching Arabic language) (2 units), physical education 2 (1 unit), pharmacology (2 units), medical sociology (2 units), general medicine 3 (3 units), general medicine 4 (3 units), preliminary vital statistics 2 (2 units), familiarity with research methods and epidemiology (2 units), computer 1 and familiarity with programming (3 units), computer 2 and familiarization with related databases and software packages (2 units), medical terms 2 (2 units), medical terms 3 (2 units), specialized language 3 (2 units), specialized language 4 (2 unit), legal aspects of medical documents (2 units), insurance and tariff of medical services and its relationship with the medical documents department (2 units), specialized medical documents archive (2 units), coding of diseases 3 (2 units), coding of diseases 4 (2 units), medical records 3 (2 units), medical records 4 (2 units), statistics and medical records seminar related to hospital issues (2 units), medical records management (2 units), internship 4 (4 units), Internship in field 5 (4 units), and internship in field 6 (4 units). Total: 66 units
		1380 = 2001	Foreign language (2 units), basic mathematics (2 units), medical degrees 1 (2 units), medical degrees 2 (2 units), anatomy (2 units), physiology (2 units), familiarity with bacteria, viruses, fungi and parasites (2 units), ethics and professional regulations (2 units), pre‐language (2 units), pre‐chemistry (2 units), hospital environment hygiene (2 units), principles of archiving method (2 units), general medicine 1 (2 unit), general medicine 2 (2 units), specialized language 1 (2 units), specialized language 2 (2 units), preliminary vital statistics 1 (2 units), physical education 1 (1 unit), Farsi (3 units), coding diseases 1 (2 units), disease coding 2 (2 units), typing (1 unit), introductory psychology (2 units), principles of hospital management (2 units), social work (1 unit), medical terminology (2 units), family planning and population (2 units), health index and hospital statistics (2 units), medical records (2 units), ethics and Islamic education (2 units), computer and its application (4 units), Islamic education 1 (2 units), internship in field 1 (4 units), internship in field 2 (4 units), and internship in field 3 (4 units). Total: 76 units
		1386 = 2007	Theoretical foundations of Islam (2 units), Islamic revolution (2 units), Islamic history and civilization (2 units), familiarity with Islamic sources (2 units), physical education 2 (1 unit), population and family planning (2 units), statistics vital (2 units), research method (2 units), medical terms 1 (2 units), medical terms 2 (2 units), health information technology (3 units), pathology 3 (2 units), pathology 4 (2 unit), pathology 5(2 units), specialized language 3(2 units), specialized language 4 (2 units), insurance and payment systems in healthcare (2 units), common systems of classification and naming of diseases and procedures 3(2 units), specialized classification systems and international nomenclature of diseases (2 units), coding of deaths (2 units), medical records 3 (2 units), medical records and their legal aspects (2 units), research project (2 units), management department of medical records (2 units), health and treatment information systems (3 units), computers and computer networks (2 units), first aid (2 units), social psychology (2 units), pharmacology (2 units), internship in the field 1 (4 units), internship in the field 2 (4 units), and internship in the field 3 (4 units). Total: 65 units
Bachelor	Health information technology	1388 = 2009	Theoretical foundations of Islam (4 units), Islamic ethics (2 units), Islamic revolution (2 units), Islamic history and civilization (2 units), familiarity with Islamic sources (2 units), physiology (2 units), anatomy (2 units), pathogenic agents and infectious diseases (2 units), mathematics (2 units), indicators and health data analysis (2 units), technology management in the field of health (2 units), specialized language (IT) 1 and 2 (4 units), data structure and computer programming (4 units), medical terms (2 units), specialized pathology 1 (2 units), epidemiology basics (2 units), health information management 1 and 2 (4 units), familiarity with operating systems and applications (2 units), management principles (2 units), pharmacology (2 units), inferential vital statistics (2 units), specialized pathology 2 and 3 (4 units), health information technology 1, 2 and 3 (7 units), insurance and payment systems in the health system (2 units), health information management in crisis (1 unit), coding of deaths (2 units), electronic health records (2 units), management of health information technology (2 units), assistance Basic (1 unit), familiarity with the structure and programs of the health system in Iran (2 units), principles of personal management and work environment (2 units), descriptive vital statistics (3 units), research methods (3 units), computer network and security systems (2 units), specialized language (HIM) 2 and 1 (4 units), management of health information resources (1 unit), specialized classification systems and international nomenclature (2 units), data quality and health information system (2 units), Research project (3 units), health informatics 1 and 2 (4 units), basics of accounting and health economics (2 units), internship in field 1 (4 units), internship in field 2 (4 units), internship in field 3 (4 units), and internship in field 4 (4 units). Total: 128 units
		1398 = 2019	Theoretical foundations of Islam (4 units), Islamic ethics (2 units), Islamic revolution (2 units), Islamic history and civilization (2 units), familiarity with Islamic sources (2 units), Persian literature (3 units), general English language (3 units), physical education 1 (1 unit), physical education 2 (2 units), family and population knowledge (2 units), history and culture and civilization of Islam and Iran (2 units), physiology (3 units), anatomy (2 units), introduction to pathogenic agents (2 units), pharmacology (2 units), basic mathematics (2 units), basic vital statistics (2 units), basics of epidemiology (2 units), computer basics (1 unit), computer basics laboratory (1 unit), research method (2 units), principles of management (2 units), medical terminology 1 (2 units), medical terminology 2 (2 units), pathology 1 (2 units), pathology 2 (2 units), pathology 3 (2 units), surgical procedures, diagnostic services, treatment and medical consumables (2 units), inferential vital statistics (2 units), HIT specific language (3 units), HIM specific language (3 units), indicators and health data analysis (2 units), basics of accounting and health economics (2 units), performance improvement management in health and treatment centers (2 units), processing and calculation of hospital insurance documents (2 units), processing and calculation of outpatient insurance documents and paraclinic (2 units), how to monitor and inspect medical centers (1 unit), health information management 1 (2 units), health information management 2 (2 units), disease coding 1 (2 units), disease coding 2 (2 units), disease coding 3 (2 units), medical procedure coding (1 unit), specialized international classifications (2 units), mortality coding (2 units), familiarity with applications (1 unit), applications laboratory (1 unit), introductory programming (1.5 units), introductory programming laboratory (0.5 units), data structures (2 units), advanced programming (1.5 units), advanced programming laboratory (0.5 units), computer networks (1.5 units), computer networks laboratory (0.5 units), databases (1.5 units), database laboratory (0.5 units), health information systems (2 units), management of health information systems (2 units), application of health information systems (2 units), application laboratory of health information systems (1 unit), analysis and display of health data (1.5 units), analysis and display of health data laboratory (0.5 units), research project (2 units) 1 and 2 health information management internship (2 units), health data analysis and indicators internship (1 unit), coding internship (1 unit), health information systems application internship (1 unit), mortality coding internship (1 unit), internship in field 1 (5 credits), and internship in field 2 (5 credits). Total: 130 units

In Table 3, the curriculum for the first chapter of bachelor's degree in HIT in 2019 consisted of 128 units, which included internships categorized as 4‐3‐2‐1 (16 units). Following a revision in 2018, the number of units increased to 130. This updated curriculum now includes 1‐2 units for health information management internships, health data indicators and analysis, coding, applications of health information systems, mortality coding, and 1‐2 field internships (16 units).

After examining the admission status of students in the field of HIT based on booklet 2 of the guide for choosing academic fields of the national exam, the experimental group of experimental sciences from 2018 to 2019 found cases as described in Table 4.

**Table 4:** Trends in student admissions for undergraduate health information technology programs in Iran (2008–present).

Year	Study period	University of Medical Sciences and nongovernmental, nonprofit higher education institute	Acceptance capacity
1388 = 2009	Daily Scholarship	Army of the Islamic Republic of Iran, Ardabil, Urmia, Isfahan, Iran, Jundishapur, Ahvaz, Zabul, Zahedan, Semnan, Shahid Beheshti, Shiraz, Kashan, Kerman, Lorestan, Mazandaran (Sari), and Mashhad.	325
1389 = 2010	Daily scholarship	Army of the Islamic Republic of Iran, Ardabil, Urmia, Isfahan, Iran, Bandar Abbas, Tabriz, Tehran, Jundishapur, Ahvaz, Zabul, Zahedan, Semnan, Shahid Beheshti, Shiraz, Kashan, Kerman, Lorestan, Mazandaran (Sari), and Mashhad.	371
1390 = 2011	Daily Scholarship	Islamic Republic of Iran, Ardabil, Urmia, Isfahan, Bandar Abbas, Tabriz, Tehran, Jundishapur, Ahvaz, Zabul, Zahedan, Semnan, Shahid Beheshti, Shiraz, Kashan, Kerman, Lorestan, Mazandaran (Sari), and Mashhad.	399
1391 = 2012	Daily Scholarship	Army of the Islamic Republic of Iran, Ardabil, Urmia, Isfahan, Bandar Abbas, Tabriz, Tehran, Jundishapur, Ahvaz, Zabul, Zahedan, Semnan, Shahid Beheshti, Shiraz, Kashan, Kerman, Lorestan, Mazandaran (Sari, Amol), and Mashhad.	491
1392 = 2013	Daily Scholarship	Army of the Islamic Republic of Iran, Ardabil, Urmia, Isfahan, Iran, Bandar Abbas, Tabriz, Torbat Haydaria, Tehran, Jundishapur, Ahvaz, Zabul, Zahedan, Semnan, Shahid Beheshti, Shiraz, Kashan, Kerman, Kermanshah, Lorestan, Mazandaran (Sari, Amol), and Mashhad.	501
1393 = 2014	Daily Scholarship	Army of the Islamic Republic of Iran, Ardabil, Urmia, Isfahan, Iran, Bandar Abbas, Tabriz, Torbat Haydaria, Tehran, Jundishapur, Ahvaz, Zabul, Zahedan, Semnan, Shiraz, Kashan, Kerman, Kermanshah, Lorestan, Mazandaran (Sari, Amol), and Mashhad.	454
1394 = 2015	Daily Self‐governing campus Excess capacity	Ardabil, Urmia, Isfahan, Bandar Abbas, Tabriz, Torbat‐Hydaria, Tehran, Jundishapur, Ahvaz, Zabul, Zahedan, Semnan, Shiraz, Kashan, Kerman, Kermanshah, Lorestan, Mazandaran (Sari, Amol), and Mashhad.	383
1395 = 2016	Daily IRGC scholarship Self‐governing campus Excess capacity	Ardabil, Urmia, Isfahan, Bandar Abbas, Birjand, Tabriz, Torbat‐Hydaria, Tehran, Jundishapur, Ahvaz, Zabul, Zahedan, Semnan, Shiraz, Kashan, Kerman, Kermanshah, Lorestan, Mazandaran (Sari, Amol), Mashhad, Abadan, and nonprofit Varastegan Mashhad.	458
1396 = 2017	Daily Self‐governing campus Excess capacity	Ardabil, Urmia, Isfahan, Bandar Abbas, Tabriz, Torbat‐Hydaria, Tehran, Jundishapur, Ahvaz, Zabul, Zahedan, Semnan, Shiraz, Kashan, Kerman, Kermanshah, Lorestan, Mazandaran (Sari, Amol), Mashhad, Hamedan, Saveh, and Mashhad nonprofit Varastegan.	467
1397 = 2018	Daily Self‐governing campus Excess capacity	Ardabil, Urmia, Isfahan, Bandar Abbas, Birjand, Tabriz, Torbat Haydaria, Tehran, Jundishapur, Ahvaz, Zabul, Zahedan, Semnan, Shiraz, Kashan, Kerman, Kermanshah, Lorestan, Mazandaran (Sari, Amol), Mashhad, Hamadan, Abadan, Saveh, neyshabour, and Varastegan Mashhad nonprofit.	558
1398 = 2019	Daily Self‐governing campus Excess capacity Native Scholarship	Army of the Islamic Republic of Iran, Ardabil, Urmia, Isfahan, Bandar Abbas, Birjand, Tabriz, Torbat Haydaria, Tehran, Jundishapur, Ahvaz, Zabul, Zahedan, Semnan, Shahroud, Shiraz, Kashan, Kerman, Kermanshah, Lorestan, Mazandaran (Sari, Amol), Mashhad, Hamadan, Yazd, Abadan, Saveh, Neishabur, and Varastegan Mashhad nonprofit.	601
1399 = 2020	Daily Tuition payer Native Scholarship	Army of the Islamic Republic of Iran, Ardabil, Urmia, Isfahan, Bandar Abbas, Birjand, Tabriz, Torbat Haydaria, Tehran, Jundishapur, Ahvaz, Zabul, Zahedan, Semnan, Shahroud, Shiraz, Kashan, Kerman, Kermanshah, Lorestan, Mazandaran (Sari, Amol), Mashhad, Hamedan, Yazd, Abadan, Saveh, Neishabur, and Varastegan Mashhad nonprofit.	597
1400 = 2021	Daily Tuition payer Native Scholarship	Army of the Islamic Republic of Iran, Ardabil, Urmia, Esfrain, Isfahan, Bandar Abbas, Birjand, Tabriz, Torbat‐Haidaria, Tehran, Jundishapur, Ahvaz, Zabul, Zahedan, Semnan, Shahroud, Shiraz, Kashan, Kerman, Kermanshah, Lorestan, Mazandaran (Sari, Amol), Mashhad, Hamadan, Yazd, Abadan, Saveh, and Neishabur and Varastegan Mashhad nonprofit.	
1401 = 2022	Daily Tuition payer Native Scholarship	Army of the Islamic Republic of Iran, Ardabil, Urmia, Esfrain, Isfahan, Bandar Abbas, Birjand, Tabriz, Torbat Haydaria, Tehran, Jundishapur, Ahvaz, Khalkhal, Zabul, Zahedan, Semnan, Shahroud, Shahrekord, Shiraz, Kashan, Kerman, Kermanshah, Lorestan, Mazandaran (Sari, Amol), Mashhad, Hamedan, Yazd, Abadan, Saveh, Neishabor, and Varastegan Mashhad nonprofit.	614
1402 = 2023	Daily Tuition payer Native Scholarship	Army of the Islamic Republic of Iran, Ardabil, Urmia, Esfrain, Isfahan, Bandar Abbas, Birjand, Tabriz, Torbat Haydaria, Tehran, Jundishapur, Ahvaz, Khalkhal, Khomein, Zabul, Zahedan, Semnan, Sirjan, Shahroud, Shahrekord, Shiraz, Kashan, Kerman, Kermanshah, Lorestan, Mazandaran (Sari, Amol), Mashhad, Hamedan, Yazd, Abadan, Saveh, Neishabor, and Varastegan Mashhad nonprofit.	654
1403 = 2024	Daily Tuition payer Native Scholarship	Army of the Islamic Republic of Iran, Ardabil, Urmia, Esfrain, Isfahan, Bandar Abbas, Birjand, Tabriz, Torbat Haydaria, Tehran, Jundishapur, Ahvaz, Khalkhal, Khomein, Zabul, Zahedan, Semnan, Sirjan, Shahroud, Shahrekord, Shiraz, Kashan, Kerman, Kermanshah, Lorestan, Mazandaran (Sari, Amol), Mashhad, Hamedan, Yazd, Abadan, Saveh, Neishabor, and Varastegan Mashhad nonprofit.	867
Total			8329

Based on the data presented in Table 4, the trend of student admissions in bachelor's program for HIT in Iran has varied since 2009, encompassing different types of admissions such as daily, scholarship, local, self‐funded campuses, excess capacity, Pasdaran scholarships, and tuition‐paying students. Initially introduced as “Bachelor of Health Information Technology,” the program later changed its name to “Health Information Technology.” Furthermore, the number of accepted students increased from 325 in 16 universities and higher education institutions in 2009 to 867 in 33 universities and institutions by 2024.

## 4 History of Graduate Programs in the Field

On February 1, 1988, a letter was sent by the head of the Medical Records subcommittee of the Supreme Cultural Revolution Council to the then Deputy Minister of Education at the Ministry of Health, highlighting the need to establish a master's program to supply faculty members and teach specialized courses. Approval was granted for this initiative, and it was instructed that the master's program should be submitted for approval to the Supreme Planning Council. Subsequently, one of the medical universities was tasked with applying for the establishment of this program at the master's level, which would officially commence upon approval from the Council for the Expansion of Medical Universities. The relevant subcommittee followed up on these steps, and this program was approved by the Supreme Planning Council of the Ministry of Culture and Higher Education on January 10, 1990.

After several decades of implementing this curriculum, and in light of the development of the field and advancements in information technology, experts and faculty members recognized the necessity for a review of the program's structure and content. They also felt it was essential to change the title from “Master's in Medical Records Education” to “Master's in Health Information Technology.” This important change was successfully executed. Additionally, a doctoral program in this field was established under the title “Health Information Management” in 1998, which has since been revised and renamed “Health Information Management”. The establishment of various university programs across Iran reflects a significant evolution in higher education, particularly in the field of health sciences. Table 5 outlines the years in which different degree programs were initiated at several medical universities in Iran.

**Table 5:** Establishment years of various university courses across Iran.

Row	Medical university	Associate degree	Bachelor of science	Master of science	PhD
1	Islamic Republic of Iran Army	1999	2009	—	—
2	Ardabil	1380	2009	1400	—
3	Urmia	1377	1387	1395	—
4	Asfarain	—	1400	—	—
5	Isfahan	1368	1376	1384	1401
6	Bandar Abbas	1996	2003	1398	—
7	Birjand	—	1397	—	—
8	Tabriz	1365	1368	1390	1395
9	Torbat Heydarieh	—	1392	—	—
10	Tehran	1366	1996	1382	2009
11	Ahvaz Jundishapur	1996	1387	1398	—
12	Khalkhal	—	1401	—	—
13	Khomein	—	1402	—	—
14	Zabol	—	2009	—	—
15	Zahedan	1380	2009	1400	—
16	Semnan	1370	2009	—	—
17	Sirjan	—	1402	—	—
18	Shahroud	—	1398	—	—
19	Shahr‐e Kord	—	1401	—	—
20	Shiraz	1363	1368	1389	1395
21	Kashan	1996	1379	2009	1395
22	Kerman	1366	1380	1392	—
23	Kermanshah	—	2009	—	—
24	Lorestan	1384	2009	1401	—
25	Mazandaran (Sari)	1369	1380	—	—
25	Mazandaran (Amol)	—	1391	—	—
26	Mashhad	1996	2009	1395	1402
27	Hamedan	—	1396	—	—
28	Yazd	—	1398	—	—
29	Abadan	—	1397	—	—
30	Saveh	1394	1396	—	—
31	Neyshabur	—	1396	—	—
32	Nonprofit Varastegan Mashhad	—	1395	—	—
33	Iran	1365	1368	1369	1377
34	Shahid Beheshti	1362	1367	1377	1382

Table 5 illustrates the timeline for the introduction of associate, bachelor's, master's, and doctoral programs at various medical universities in Iran. The establishment of these programs began in the late twentieth century and has continued into the present day, showcasing a commitment to expanding educational opportunities in health‐related fields.

The data indicates that universities such as Isfahan and Tehran have a long history of offering diverse programs, while newer institutions are emerging to meet contemporary educational demands. The growth in the number of programs reflects not only an increase in student enrollment but also a response to the evolving needs of the healthcare sector in Iran.

In summary, the progression of graduate programs across Iranian universities highlights a significant development in medical education, aiming to enhance the quality of healthcare services through well‐trained professionals. This historical overview provides insight into how higher education has adapted to societal needs and technological advancements over time.

Approved educational programs for different fields of study in Iran

The educational programs approved by the Higher Planning Council of Medical Sciences within the Ministry of Health, Treatment, and Medical Education in Iran are outlined in the following Table 6.

**Table 6:** List of approved educational programs as of September 2024.

Academic levels	Field of study
Associate degree	Medical records
Bachelor's degree	Health information technology (continuous)
	Medical records (discontinuous)
Master's degree (noncontinuous)	Health information technology
PhD	Health information management

This organized overview highlights the different educational pathways available in the medical sciences, demonstrating the progression from associate to doctoral levels within specific fields. The inclusion of both continuous and discontinuous bachelor's programs reflects a flexible approach to education, catering to diverse student needs and career aspirations. The emphasis on HIT and management indicates a growing recognition of the importance of these fields in enhancing healthcare delivery and administration in Iran.

Currently, students enrolled in the associate degree program for medical records are being accepted at the Iranshahr Faculty of Medical Sciences, as outlined in Table 7.

**Table 7:** Summary of admission processes and program information for medical documents associate degree.

Course type	Admission method	University name	Province	Field of study	Gender	Capacity
Daily	Without exam, semi‐centralized	Iranshahr School of Medical Sciences	Sistan and Baluchestan	Associate degree in medical documents	Female, male	20

Graduates in health information technology who wish to further their education have the opportunity to pursue master's and doctoral degrees. Below are examples of specializations available for those holding a bachelor's degree in this field:

– Health information technology;
– Health technology assessment;
– Medical engineering (including biomedical and biomaterials);
– Medical biotechnology;
– Epidemiology;
– Health economics;
– Medical informatics;
– Health education;
– Medical librarianship and information science;
– Healthcare services management;
– Medical nanotechnology;
– Social welfare;
– History of medical sciences;
– Biostatistics;
– Medical education;
– E‐learning planning in medical sciences;
– Medical journalism;
– Educational technology in medical sciences.

These specializations provide a range of options for graduates seeking to advance their knowledge and skills in various aspects of health information and related disciplines.

## 5 Discussion

In the late 1980s and early 1990s, the affordability, power, and compactness of hardware significantly improved, leading to the widespread use of personal computers, local area networks, and the Internet. This evolution facilitated faster and easier access to medical information and initiated the adoption of Web‐based EHRs [24]. An initial challenge in utilizing EHRs was the requirement for portable computing devices [25], however, this issue was soon addressed as computers evolved into various forms, including laptops, palmtops, notebooks, and pen computers [26].

With the rapid advancement of technology and the growing demand for related fields, the terminology surrounding this profession shifted globally from “medical records” to “HIT.” This change aimed to establish a contemporary framework for managing student information in health and ensuring safe information exchange.

The planning of educational programs in HIT is closely tied to the rapid advancement of new computer technologies. This relationship is evident in the continually evolving curriculum in this area. Researchers have worked to identify and clarify these changes by examining existing documents. To fill the current gaps, it is crucial to gather input from industry professionals and educators during curriculum revisions. Future enhancements might include the creation of standardized training programs that guarantee all students receive a thorough education, irrespective of their location. Furthermore, investigating international best practices in HIT education could offer valuable perspectives for improving programs in Iran.

Additionally, it is important to consider the implications for other countries that encounter similar challenges in HIT education. Collaborative initiatives and knowledge sharing can strengthen a global strategy for training professionals in this vital sector.

While the curriculum does not explicitly mention AI or blockchain, these technologies are integrated into the HIT education through various courses. Instructors are allowed to dedicate up to 20% of each course to essential topics, including AI and blockchain applications in healthcare. Additionally, practical training sessions provide students with hands‐on experience using tools based on these technologies, enhancing their understanding of real‐world applications.

To address this oversight, the discussion will be expanded to include postgraduate and doctoral education. This expansion will highlight how advanced degrees incorporate emerging technologies like AI and blockchain, as well as the unique challenges and opportunities they present in the field of HIT. This insight will enhance the comprehensiveness of the work.

It is appreciated that the current version primarily emphasizes undergraduate programs in HIT education. To enhance the comprehensiveness of the work, a section will be added that addresses postgraduate and doctoral education. This addition will explore how advanced degree programs incorporate emerging technologies such as AI and blockchain, as well as the unique challenges and opportunities they present in the HIT field

Finally, concerning ethical considerations, no ethical issues were identified in this study; a statement addressing conflicts of interest and ethical guidelines has been provided to ensure transparency.

Consider incorporating the following elements into your article:

## 6 Introduction of Ethical Challenges

- Cultural Context: Begin by discussing the unique cultural landscape of Iran and how it influences HIT education. Highlight specific cultural values or norms that may affect students' perceptions of technology and ethics.
- Ethical Frameworks: Reference relevant ethical frameworks that apply to HIT education, such as Islamic ethical principles, and discuss how these can be integrated into the curriculum.

## 7 Analysis of Current Curriculum

Comparison With Other Curricula: Analyze how the Iranian HIT curriculum compares to those in other countries, especially in terms of addressing ethical issues. For instance, you could reference findings from studies on moral education in Iranian schools that emphasize the need for alignment with both national and multicultural educational standards [27].

Deficiencies Identified: Discuss any identified gaps in the current curriculum regarding ethical training and cultural considerations. For example, you might draw from qualitative studies that reveal challenges faced in medical ethics education in Iran [28].

## 8 Proposed Strategies for Improvement

- Curriculum Development: Suggest specific strategies to enhance the HIT curriculum by incorporating ethical training that reflects both local cultural values and global standards. This could include:
  ○ Integrating case studies that reflect local ethical dilemmas.
  ○ Providing professional development for educators on ethical issues relevant to HIT.
  ○ Creating interdisciplinary courses that blend technology with ethics and cultural studies [29].

### 8.1 Engagement With Stakeholders

It is recommended that a diverse group of stakeholders, including students, instructors, and health professionals, be involved in curriculum design to develop a program that addresses the needs of all parties while respecting cultural differences and characteristics.

### 8.2 Future Research Directions

Encourage further research into how HIT education can adapt to evolving ethical challenges brought about by advancements in technology. This could include exploring how other countries successfully integrate cultural considerations into their HIT curricula.

After presenting your findings or results, transition into a discussion about how these findings relate to the broader context of HIT education and its role in addressing healthcare challenges in Iran.

## Author Contributions

**Azita Balaghafari:** conceptualization, investigation, funding acquisition, writing – original draft, writing – review and editing, visualization, validation, methodology, project administration, formal analysis, software, resources, supervision, data curation. **Hasan Siamian:** conceptualization, investigation, funding acquisition, writing – original draft, methodology, validation, writing – review and editing, visualization, project administration, formal analysis, software, data curation, supervision, resources.

## Ethics Statement

The study was approved by the Ethical Committee of Mashhad University of Medical Sciences with Ethical number of the IR. NASRME. REC.1402.017.

## Conflicts of Interest

The authors declare no conflicts of interest.

## Transparency Statement

The lead author Hasan Siamian affirms that this manuscript is an honest, accurate, and transparent account of the study being reported; that no important aspects of the study have been omitted; and that any discrepancies from the study as planned (and, if relevant, registered) have been explained.

## Data Availability

All data generated or analyzed during this study are included in this published article. All authors have read and approved the final version of the manuscript, had full access to all of the data in this study, and take complete responsibility for the integrity of the data and the accuracy of the data analysis. The data that support the findings of this study are available from the corresponding author (Hasan Siamian), upon reasonable request.
